# Influence of blue-light blocking filter on a series of diagnostic tasks in digital radiographs

**DOI:** 10.1007/s11282-026-00949-z

**Published:** 2026-07-14

**Authors:** Julia Beatriz Tonon, Gabriel Luiz Freire Torres, Julia Moreira Biokino, Michelle Chang, Marcos Paulo Maia-Lima, Deborah Queiroz Freitas, Amanda Farias-Gomes, Hugo Gaêta-Araujo

**Affiliations:** 1 Faculdade de Odontologia de Ribeirão Preto, Universidade de São Paulo, Ribeirão Preto, Brazil; 2 Faculdade deOdontologia de Piracicaba, Universidade Estadual de Campinas, Piracicaba, Brazil; 3 Faculdade de Odontologia, Universidade de São Paulo, São Paulo, Brazil

**Keywords:** Blue light filtration, Digital radiography, Diagnostic imaging, Dental

## Abstract

**Objectives:**

To evaluate the influence of blue-light filtering on the diagnosis of different dental conditions (internal and external root resorptions, horizontal and vertical root fractures, and furcation defects) in intraoral digital radiographs.

**Methods:**

The study used 10 mandibles, 8 dry skulls, and 60 single-rooted teeth distributed according to the induction of the different pathological conditions. The conditions were simulated using specific methodologies for each type of lesion with mechanical processes or combined with chemical processes. Digital radiographs were acquired using the KaVo eXam digital system. The obtained images were randomized and evaluated individually by 11 evaluators, using a 5-point scale, as to the absence or presence of each condition. The images were evaluated using Windows 11 blue-light filtering with four intensities: 0% (off), 20% (low), 40% (intermediate), and 60% (high). Diagnostic values were calculated and compared using one-way ANOVA with a 5% significance level.

**Results:**

There were no significant differences in the diagnostic values among the different intensities of the blue-light filtering for any of the diagnostic tasks (*p* ≥ 0.05).

**Conclusions:**

The use of blue-light filtering at different intensities does not influence diagnostic tasks on digital radiographs. Thus, this resource can be used by professionals.

## Introduction

Technology has integrated into both personal and professional life, offering quick access to information and enhancing convenience [1]. The widespread use of digital systems and prolonged screen time in recreational, educational, and professional settings has led to dependence of individuals on these devices, contributing to greater eye fatigue [2].

Despite the benefits of digital technology, increased screen exposure has changed how human eyes are exposed to light 3^3^. Prolonged screen use has led to greater exposure of professionals to blue-light, a high-energy, short-wavelength (400–500 nm) light emitted in greater proportions by screens than by traditional light sources [3, 4]. According to the literature, blue light can affect physiological functions, as it suppresses the secretion of melatonin hormone, altering the circadian rhythm and sleep-wake cycle [5, 6]. It may also promote DNA damage, cell death, photoaging [7], retinal phototoxicity [8, 9] and increase the risk of cataracts and age-related macular degeneration due to the accumulation of toxic oxygen radicals [10, 11].

To prevent the harmful effects of prolonged blue light exposure, various devices have been proposed, such as blue-light blocking ophthalmic lenses [12], sepia mode on computers and tablets, as well as apps and settings designed in Windows^®^ and Android^®^ operating systems that filter blue light [13]. These tools modify visual input by blocking or attenuating specific wavelengths of light [14], typically by decreasing the screen luminance and adding a yellow chromophore, which helps protect the users from blue-light exposure and seems to reduce visual fatigue [4].

In the dental field, digitalization has advanced rapidly in recent years [15]. Among the developed digital technologies, digital radiology can be highlighted, which includes the replacement of traditional radiographic films with digital receptors. Advantages of digital radiography include faster image acquisition (eliminating the need for chemical process), real-time display (for sensors), the possibility to apply post-processing enhancement filters to the image, and reduced radiation exposure to the patient due to higher sensitivity of the receptors [16, 17]. Additionally, digital radiographs can be visualized on computer screens, tablets, and smartphones, without the need for printing and facilitating image sharing [18, 19].

Radiologists may be more vulnerable to developing pathologies related to excessive exposure to blue light, as they spend hours in front of computer screens interpreting radiographic images [4] and working on challenging diagnoses such as internal and external root resorptions [20], vertical and horizontal root fractures [21], and furcation defects [22]. Diagnosing these lesions is challenging, as radiographs are two-dimensional images representing three-dimensional structures, which can lead to overlapping anatomical structures and limit their evaluation [23].

Since previous studies have shown that blue-light filters may help reduce eye fatigue symptoms [4], and given their widespread availability, professionals with prolonged blue-light exposure may already use them or could benefit from doing so. However, these filters generally increase the warmth of the device’s screen, resulting in a more yellowish appearance [9] and reducing color contrast sensitivity [24]. Although radiographic images are presented in grayscale instead of different colors, it is reasonable to assume that the perception of radiographic image contrast could also be impacted by the use of blue-light filters. This concern is particularly relevant in dentistry, where radiographic diagnosis frequently relies on subtle perception of contrast between shades of gray. Conditions such as internal and external root resorptions, root fractures, and furcation defects often presents as discrete changes in radiopacity that demand fine grayscale discrimination. While prior research has investigated the effects of blue-light filters on visual fatigue and eye health [4], none have specifically assessed whether these filters interfere with diagnostic performance. Therefore, it is important to evaluate whether the use of blue-light filters interfere in the radiographic diagnosis of different pathological conditions.

Therefore, the aim in this study was to evaluate the influence of different intensities of the blue-light blocking filter (i.e., “Night Light” mode) available in the Windows^®^ 11 operating system (Microsoft Corporation, Redmond, USA) on the diagnosis of internal and external root resorptions, horizontal and vertical root fractures, and furcation defects in digital intraoral radiographs. The null hypothesis considered that the application of different intensities of the blue-light blocking filter would not influence the diagnosis of the evaluated conditions.

## Methods

This study was approved by the local Research Ethics Committee (protocol number CAAE: 67720423.6.0000.5419) and in accordance with the Helsinki Declaration. The samples were composed of 30 single root canines and premolars used for internal root resorption (IRR) and external root resorption (ERR) inductions; 15 lower incisors, canines, and premolars for vertical root fractures (VRF) and 15 upper incisors for horizontal root fractures (HRF) inductions; and 18 molars present in dry mandibles (*n* = 10) and skulls (*n* = 8) for furcation defects (FD) inductions.

### Sample preparation

All the teeth of the study samples were disinfected with 70% alcohol and cleaned to remove calculus and tissue remnants. The teeth were maintained in water until used.

### Root resorption (RR)

The crowns of 30 teeth were sectioned at the cement-enamel junction using an Isomet diamond blade (Isomet 1000, Buehler Ltd., Lake Bluff, USA). For IRR simulation, 10 teeth were sectioned longitudinally in the mesio-distal direction into two halves using an Isomet diamond blade (Isomet 1000, Buehler Ltd., Lake Bluff, USA). The IRR lesions were induced using a combination of mechanical process using spherical diamond burs (KG Sorensen, Cotia, Brazil) of size 1 and chemical process involving demineralization with 5% nitric acid and 8% sodium hypochlorite solutions for 24 h [25]. The two halves of each tooth were re-attached using a thin layer of cyanoacrylate-based adhesive (Scotch Super Glue, 3 M, Maplewood, MN, USA). For ERR, the lesion was simulated in a restricted area of the apical third of the dental roots of another 10 teeth, following the methodology proposed by Kolsuz et al. (2022) [26]. In addition to the teeth with simulated resorptions (i.e., 20 teeth), 10 teeth without lesions induction (control group) were included in the sample for this diagnostic task. All samples with simulated lesions were visually inspected to confirm the presence of resorption.

### Root fractures (RF)

For the VRF induction, the crowns of 10 teeth were sectioned at the cement-enamel junction using an Isomet diamond blade (Isomet 1000, Buehler Ltd., Lake Bluff, USA) and were subjected to root canal instrumentation following the MTwo rotary protocol (VDW, Munich, Germany). Then, the teeth were subjected to fracture induction by inserting a blunt metal tip into the root canal entrance and applying a compressive force of 1 mm/min by using a universal testing machine (Instron Corporation, Norwood, MA, USA). For the HRF induction, a horizontal incision was created in the middle third of 10 teeth using an IsoMet^®^ high-precision cutter (Buehler, Illinois, USA) and a 0.3 mm diamond blade. The separated fragments were reattached using a thin layer of cyanoacrylate-based adhesive (Scotch Super Glue, 3 M, Maplewood, MN, USA). In addition to the teeth with simulated fractures (i.e., 20 teeth), 10 teeth without simulated lesions (control group) were included in the sample for this diagnostic task. The simulated conditions were confirmed using visual inspection and transillumination.

### Furcation defects (FD)

A grade 3 FD was simulated with the removal of the buccal bone adjacent to the teeth, exposing the furcation region and establishing communication between the buccal and lingual/proximal surfaces [27]. The defects were created by restricting the wear only to the bone tissue, using spherical and conical diamond burs (KG Sorensen, Cotia, Brazil) of sizes ½ to 4 compatibles with the anatomy of each tooth. The process was performed under water irrigation, using diamond burs attached to high-speed handpiece, with progressive removal in 1 mm increments to prevent excessive bone loss. Images of the teeth before FD induction were acquired to be included in the sample as teeth without bone loss (control group). The simulated conditions were confirmed by probing.

### Image acquisition

The KaVo eXam digital radiographic system (KaVo, Tuusula, Finland) with size 2 phosphor plates was used for image acquisition. For all the samples, the intraoral X-ray machine used was the Spectro 70X (Dabi Atlante, Ribeirão Preto, Brazil) and the acquisition parameters were set at 70 kVp, 8 mA and 0.28 s of exposure.

The focus-receptor distance was standardized at 40 cm, using an acrylic device to reproduce the parallelism technique and allow standardization and reproducibility of the acquired images. In addition, a 2.5 cm thick acrylic block was positioned between the X-ray tube and the samples to simulate the dispersion and attenuation of the X-ray beam by the soft tissues. The images were obtained with an orthoradial incidence (i.e., with the radiation beam perpendicular to the region to be radiographed), following the orientation of the acrylic device’s orientation ring.

**Fig. 1:**
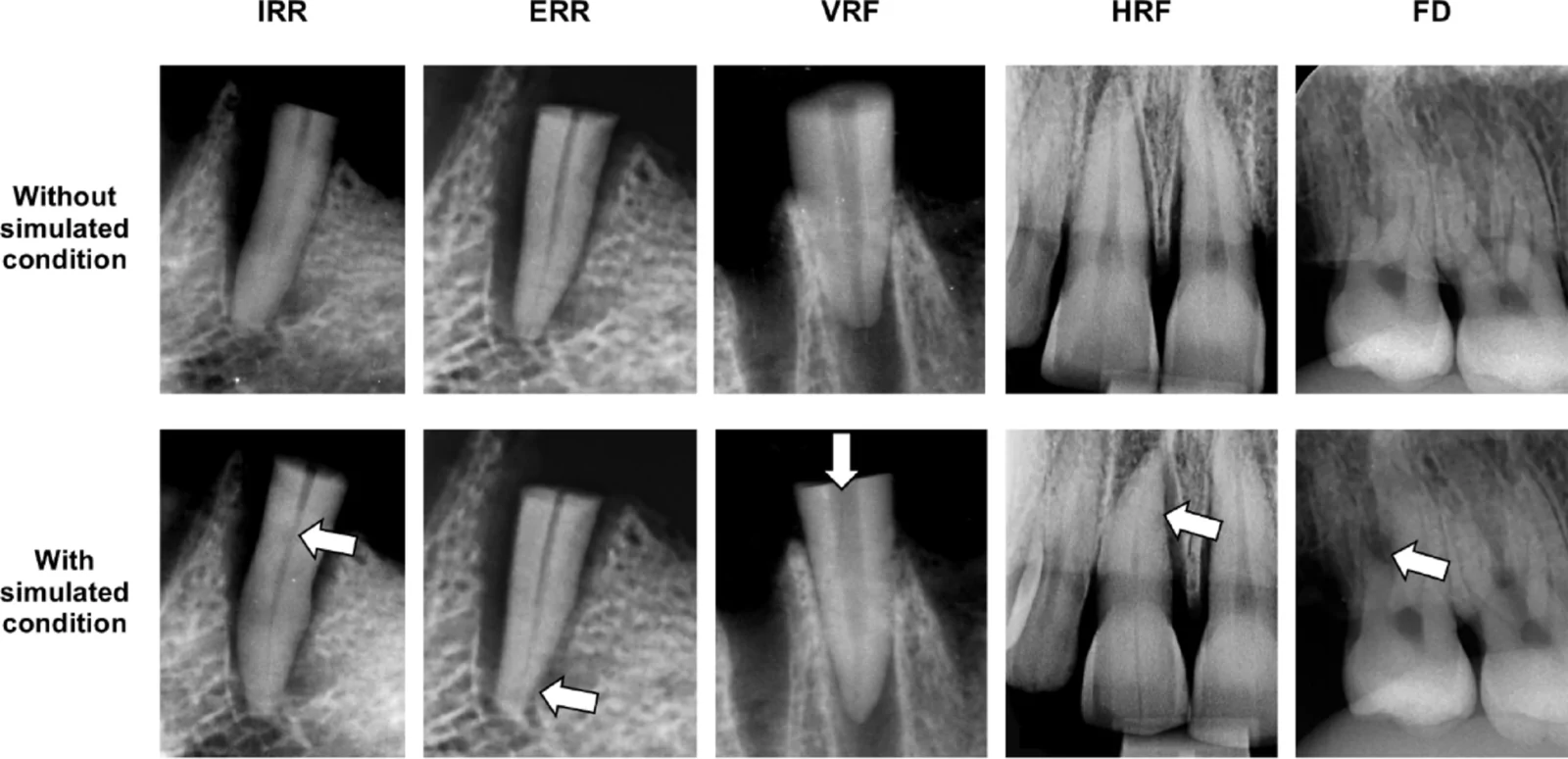
Images of the groups without simulated condition and with induction of five pathology conditions: IRR, ERR, VRF, HRF, and FD

To acquire the radiographic images of the samples corresponding to tasks: RR and VRF, each tooth (with and without the conditions) was placed individually into the dental alveolus of a dry human mandible corresponding to tooth 45 (Fig. 1). For the acquisition of HRF, the teeth were positioned into the dental alveolus of a dry human skull corresponding to tooth 11. To acquire the samples for the task corresponding to FD, each skull or mandible was positioned on an acrylic device and stabilized with molding material to standardize the image acquisitions. The radiographic acquisitions were made at two moments: before the induction of FD, representing the group of images without bone loss (i.e., control group) and after the induction of grade 3 FD (Fig. 1).

### Image evaluation

**Fig. 2:**
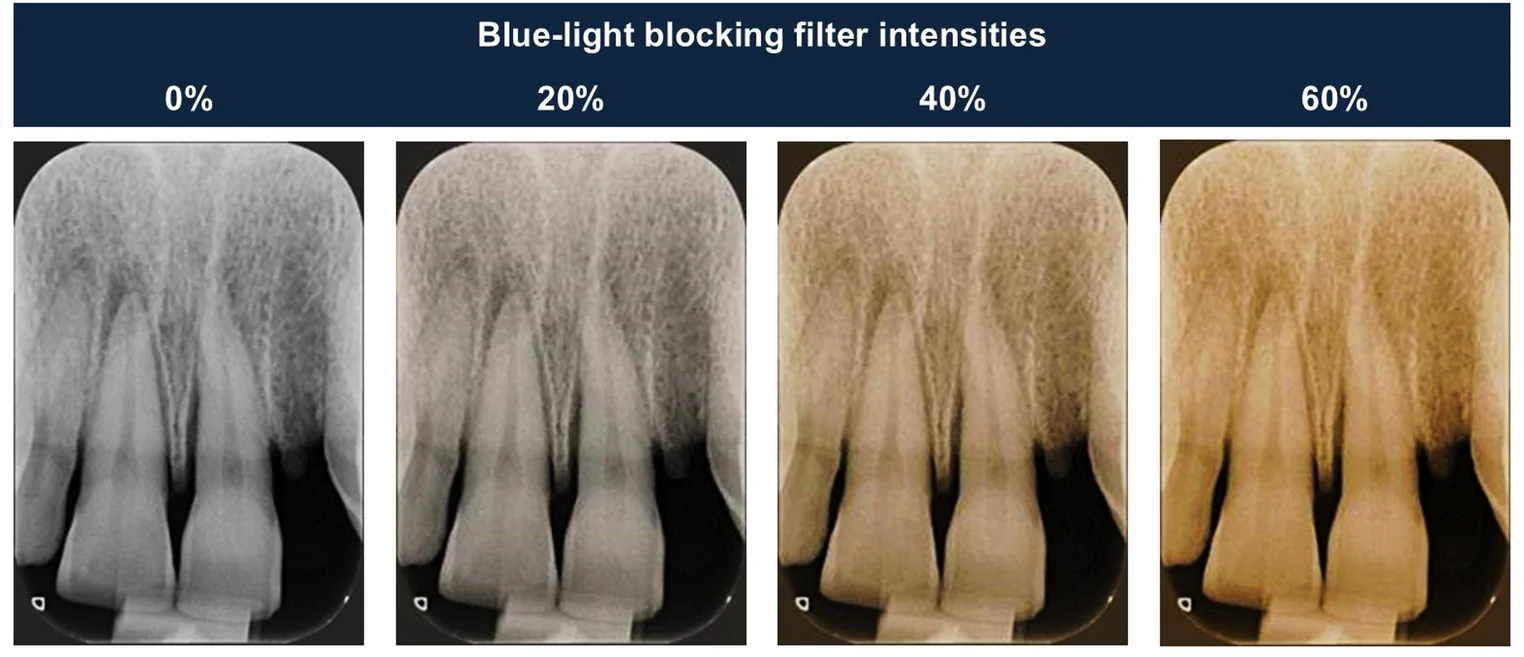
Images showing the screen appearance at the different intensities of blue-light filtering: 0%, 20%, 40%, and 60%

The images were exported in TIFF format with 8-bit contrast resolution. Each different condition (i.e., IRR, ERR, VRF, HRF, and FD) was assessed at different sessions by the examiners. For each condition, the images were randomly organized for 4 sessions of evaluation by renaming the files according to a random order generated by the website www.random.org. For each session of each condition, the Windows 11 blue-light filtering (Microsoft Corporation, Redmond, WA, USA), in “Night Light” mode, was set at different intensities: 0% (off), 20% (low), 40% (intermediate), and 60% (high) (Fig. 2). It is worth noting that the Night Light mode operates by shifting the screen color temperature toward warmer tones rather than selectively filtering only blue wavelengths. The term ‘blue-light filter’ is used throughout this manuscript in accordance with the terminology most commonly adopted in the clinical and scientific literature. According to Microsoft’s official documentation [25], the Night Light mode adjusts the screen color temperature toward warmer tones, with the strength scale ranging from 0% (6500 K – off) to 100% (1200 K – maximum intensity). The exact Kelvin values for intermediate steps are not publicly disclosed by Microsoft and may vary slightly depending on monitor hardware and drivers. Based on this scale, the four intensity levels tested in this study correspond approximately to 6500 K (0%), 5200 K (20%), 4400 K (40%), and 3350 K (60%). The Adaptive Color feature of Windows 11 was confirmed to be disabled during all evaluation sessions, and the tested monitor does not incorporate an ambient light sensor, ensuring that the only color temperature modification applied was the one deliberately set by the Night Light mode at each tested intensity. Therefore, there were 4 sessions for each condition, totaling 20 sessions. There was an interval of a week between each session to avoid memorization.

Eleven oral radiologists were invited to evaluate the images individually, using a 5-point scale, according to the presence or absence of the assessed diagnostic task: (1) absence of lesion; (2) probable absence of lesion; (3) uncertain diagnosis; (4) probable presence of lesion, and (5) presence of lesion. The images were evaluated in a room with controlled lighting, using the JPEGView 1.0.35.1 software (public domain software), on a high-resolution monitor (MDRC-2124, Barco N.V., Courtray, Belgium), which is DICOM Part 14 compliant and equipped with Backlight Output Stabilization (BLOS) technology, ensuring consistent and stable luminance output. The examiners could adjust brightness and contrast and use zoom tool at their discretion. To prevent visual fatigue of the evaluators, the assessments were organized so that only one session was conducted per day. Thirty days after the end of the assessments, 20% of the sample of each condition was randomly re-evaluated to verify the intra-examiner reproducibility.

### Statistical analysis

The data was analyzed using SPSS Version 25.0 (IBM SPSS Statistics for Windows, Armonk, NY, USA) and GraphPad Prism Version 8.0 (GraphPad, La Jolla, CA, USA), with a 5% significance level. The intra- and inter-examiner agreements were assessed using the weighted Kappa and interpreted according to the Landis & Koch (1977) scale: <0.00 (poor), 0.00-0.20 (slight), 0.21–0.40 (fair), 0.41–0.60 (moderate), 0.61–0.80 (substantial), and 0.81-1.00 (almost perfect).

The area under the Receiver Operating Characteristic (AUROC) curve was calculated from the 5-point scale and the accuracy, sensitivity, and specificity values were calculated from the dichotomization of the points, considering scales 1 and 2 as absence of the condition and 3, 4, and 5 as presence of the condition. These values were calculated separately for each evaluator and each diagnosis, considering each interpretation condition (i.e., intensity of the blue-light filter) and expressed as mean and standard deviation (SD) of the evaluators. Diagnostic values were compared using one-way ANOVA with Tukey’s post-hoc test. The significance level was established at 5% (α = 0.05).

## Results

### Root resorptions

The mean intra-examiner agreement values were 0.282 (fair agreement) and 0.209 (fair agreement) for IRR and ERR, respectively. The mean inter-examiner agreements were 0.182 (slight agreement) and 0.180 (slight agreement) for IRR and ERR, respectively.

Mean and SD of the AUROC, accuracy, sensitivity, and specificity values for the detection of IRR and ERR considering the application of different intensities of blue-light blocking filter are shown in Table 1. There were no significant differences (*p* ≥ 0.05) among the different intensities of the blue-light blocking filter for the diagnosis of neither IRR nor ERR.

**Table 1:** Mean (SD) AUROC, accuracy, sensitivity, and specificity values for IRR and ERR detection considering application of different intensities of blue-light filtering

Intensities of blue-light filtering	Diagnostic values – IRR and ERR Mean (SD)							
	AUROC		Accuracy		Sensitivity		Specificity	
	IRR	ERR	IRR	ERR	IRR	ERR	IRR	ERR
Filter 0%	0.75 (0.13)	0.60 (0.09)	0.69 (0.13)	0.55 (0.08)	0.56 (0.20)	0.56 (0.18)	0.81 (0.21)	0.55 (0.28)
Filter 20%	0.66 (0.12)	0.54 (0.06)	0.63 (0.13)	0.52 (0.07)	0.45 (0.19)	0.50 (0.24)	0.81 (0.21)	0.54 (0.26)
Filter 40%	0.72 (0.09)	0.50 (0.08)	0.64 (0.07)	0.50 (0.09)	0.49 (0.21)	0.53 (0.02)	0.80 (0.17)	0.48 (0.30)
Filter 60%	0.75 (0.08)	0.56 (0.13)	0.67 (0.07)	0.53 (0.12)	0.49 (0.14)	0.49 (0.22)	0.86 (0.16)	0.58 (0.29)

Note: There were no statistically significant differences between the different intensities of blue-light filtering (*p* ≥ 0.05)

*AUROC* area under the receiver operating characteristic curve, *IRR* internal root resorption, *ERR* external root resorption

### Root fractures

The mean intra-examiner agreement values were 0.483 (moderate agreement) and 0.468 (moderate agreement) for VRF and HRF, respectively. The mean inter-examiner agreement values were 0.375 (fair agreement) and 0.389 (fair agreement) for VRF and HRF, respectively.

Mean and SD of the AUROC, accuracy, sensitivity, and specificity values for assessing VRF and HRF considering the application of different intensities of blue-light blocking filter are shown in Table 2. There were no significant differences (*p* ≥ 0.05) among the different intensities of the blue-light blocking filter for the diagnosis of neither VRF nor HRF.

**Table 2:** Mean (SD) AUROC, accuracy, sensitivity, and specificity values for VRF and HRF detection considering application of different intensities of blue-light filtering

Intensities of blue-light filtering	Diagnostic values – VRF and HRF Mean (SD)							
	AUROC		Accuracy		Sensitivity		Specificity	
	VRF	HRF	VRF	HRF	VRF	HRF	VRF	HRF
Filter 0%	0.66 (0.09)	0.74 (0.07)	0.54 (0.14)	0.66 (0.13)	0.73 (0.11)	0.75 (0.16)	0.64 (0.11)	0.82 (0.11)
Filter 20%	0.69 (0.05)	0.75 (0.06)	0.57 (0.08)	0.67 (0.11)	0.80 (0.17)	0.74 (0.21)	0.65 (0.17)	0.85 (0.13)
Filter 40%	0.68 (0.07)	0.73 (0.08)	0.56 (0.11)	0.61 (0.13)	0.81 (0.12)	0.76 (0.20)	0.66 (0.22)	0.79 (0.13)
Filter 60%	0.64 (0.11)	0.69 (0.13)	0.51 (0.15)	0.59 (0.18)	0.71 (0.12)	0.68 (0.22)	0.64 (0.17)	0.79 (0.21)

Note: There were no statistically significant differences between the different intensities of blue-light filtering (*p* ≥ 0.05)

*AUROC* area under the receiver operating characteristic curve, *VRF* vertical root fracture, *HRF* horizontal root fracture

### Furcation defects

The mean intra-examiner agreement value was 0.434 (moderate agreement) and 0.296 (fair agreement) for inter-examiner agreement for FD detection.

Mean and SD of the AUROC, accuracy, sensitivity, and specificity values for assessing FD considering the application of different intensities of blue-light blocking filter are shown in Table 3. There were no significant differences (*p* ≥ 0.05) among the different intensities of the blue light blocking filter for the diagnosis of FD.

**Table 3:** Mean (SD) AUROC, accuracy, sensitivity, and specificity values for FD detection considering application of different intensities of blue-light filtering

Intensities of blue-light filtering	Diagnostic values – FD Mean (SD)			
	AUROC	Accuracy	Sensitivity	Specificity
Filter 0%	0.81 (0.09)	0.76 (0.08)	0.73 (0.12)	0.80 (0.15)
Filter 20%	0.83 (0.09)	0.79 (0.08)	0.73 (0.14)	0.85 (0.13)
Filter 40%	0.82 (0.08)	0.76 (0.07)	0.71 (0.12)	0.81 (0.12)
Filter 60%	0.81 (0.08)	0.75 (0.07)	0.71 (0.14)	0.79 (0.16)

Note: There were no statistically significant differences between the different intensities of blue-light filtering (*p* ≥ 0.05)

*AUROC* area under the receiver operating characteristic curve, *FD* furcation defect

## Discussion

Some benefits of blue-light blocking filters to users, including improved sleep quality and decreased cortisol levels [28], reduced eye strain, and alleviation of other eye fatigue symptoms such as headaches and blurred vision [4], have been reported by studies in different areas. However, these filters increase the warmth or yellowing of the device’s screen by decreasing its luminance and adding a yellow chromophore when activated [9]. Thus, our hypothesis was that a blue-light filter could impact the perception of gray tones on radiographic images and thereby compromise the diagnosis. Nevertheless, according to the obtained results, there was no influence of the application of a blue-light blocking filter on the radiographic diagnosis of different dental conditions, even when the filter with high-intensity (i.e., 60%) was applied. Therefore, the proposed null hypothesis was accepted.

Although the efficacy of blue-light filters in reducing eye fatigue remains debated in the literature, some studies have shown their benefit in alleviating symptoms [4], or at least in providing visual comfort to screen users. A pilot study involving radiology residents has found that blue-light filtering lenses ameliorated the symptoms of computer vision syndrome (CVS), which includes eyestrain, headaches, blurred vision, and dry eyes [4]. Given these findings and the widespread commercialization of blue-light filters for this purpose, it seemed reasonable to assume that radiologists might be using them during image evaluation. Therefore, it was necessary to assess whether these filters affect diagnostic capacity in different radiographic tasks with varied radiographic appearances. The diagnostic values obtained in our study for all filter conditions (i.e., no filter and various blue-light filter intensities) were similar to those reported by previous studies on the same diagnostic tasks, though conducted in different contexts [21, 29–31].

Regarding the diagnosis of IRR and ERR, no significant differences for the conditions of blue-light filter were observed for either of them. It was noted that both the study conducted by Oliveira-Santos et al. (2022) [30] and our research found that diagnostic values for ERR were lower than those for IRR. This difference can be attributed to the distinct radiographic appearance of these lesions: IRR typically appears as a diffuse radiolucent area within the root canal, surrounded by the radiopaque image of dentin, while ERR presents as a diffuse contour change on the root surface, which can make detection more challenging.

In relation to the RF assessment, our results showed that the application of the blue-light blocking filter at different intensities had no significant effect on either VRF or HRV. Lower diagnostic values were found for VRF compared to HRF, which is probably related to the greater challenge in diagnosing VRF [32]. Also, although not statistically significant, lower diagnostic values were observed for both types of RF when the filter was applied at its maximum intensity. This may be explained by the excessive yellowing in the image, which may have compromised the perception of the subtle contrast between the fracture line and the dentin.

As with the other assessed diagnostic tasks, the blue-light filter at different intensities had no impact on the diagnostic values for FD. A previous study on this task but evaluating the effect of different spatial resolutions and enhancement filters on FD detection in intraoral radiographs [31], obtained diagnostic values similar to ours (0.815 for AUROC and around 0.760 for accuracy, sensitivity, and specificity), supporting our findings. Also, our study showed low intra- and inter-examiner agreements, with average values of 0.434 and 0.296, respectively. Low agreement values were also demonstrated by Santos et al. (2023) [31] for the same diagnostic task. The difficulty of the diagnostic task and the inclusion of upper molars – in which the palatal root often overlaps the region of interest in radiographs – in the sample, likely contributed to these low agreement values.

Ex-vivo studies with simulated conditions have limitations compared to real clinical settings. However, the methods used to obtain images were designed to closely replicate clinical conditions, using human skulls, mandibles, and teeth, along with acrylic to simulate the X-rays attenuation by soft tissues. Also, despite the inherent limitations, the ex vivo setup offers advantages, such as the possibility to calculate accuracy, sensitivity, and specificity for the tested diagnostic tasks, which require a known reference standard – something that may not always be feasible in clinical cases. Additionally, it would not be ethically acceptable to expose patients to radiation solely for research purposes, a fact mitigated by the use of an ex vivo methodology.

A strong aspect of the present study was the inclusion of 11 examiners, which enhances the generalizability of our findings. The image evaluations were organized into sessions based on the condition and filter intensity being analyzed. Each session typically lasted around 30 min, and examiners could choose the time of day that suited their schedule. However, considering that blue-light blocking filters may be effective in reducing the symptoms of eye fatigue, further studies should investigate their impact on radiographic diagnosis at the end of a working day.

The present study tested the digital blue-light blocking filter available in the Windows operating system, known as “Night Light”. Nevertheless, other resources to block the blue-light wavelength, such as glasses, lenses, and screen covers, are also available and should be further explored to assess their impact on balancing professional work quality and diagnostic performance. Our main hypothesis was that blue-light filters could hinder digital radiographic diagnosis, as their mechanism reduces screen luminance and adds a yellow chromophore [9]. However, considering that these filters may allow the professional’s work to be optimized by improving sleep quality and preventing the suppression of cortisol release caused by blue-light [28], further studies should investigate their potential positive impact on performance. Furthermore, although none of the evaluators in the present study had color vision deficiency (CVD), this variable should be considered in future research. Although CVD is primarily associated with the inability to differentiate certain colors, and our study used radiographic images presented in grayscale, it cannot be entirely excluded that this condition could potentially alter image perception and therefore influence diagnostic performance, particularly given that blue-light filters modify screen color temperature [34].

The use of digital screens has become a daily habit for many individuals, being used for work, entertainment, and various other functions. Currently, chemical processing of radiographs has become outdated, and radiographic images are mostly available digitally, making monitors indispensable. The lack of differences among the tested blue-light filter conditions (no filter and different intensities of filter) for different diagnoses suggests that the filter does not substantially alter the radiopacity and radiolucency of the images and therefore does not influence the final radiographic diagnosis. Based on our findings, the tested filter at the used intensities can be employed without affecting diagnostic performance. Therefore, this resource, which is available on any Windows computer, may be recommended for use by professionals.

## Conclusions

The blue-light blocking filter tested at different intensities (up to 60%) does not influence the diagnosis of IRR, ERR, VRF, HRF, and FD. This resource can be used by professionals according to their preference.

## Data Availability

No datasets were generated or analysed during the current study.
